# So depression is an inflammatory disease, but where does the inflammation come from?

**DOI:** 10.1186/1741-7015-11-200

**Published:** 2013-09-12

**Authors:** Michael Berk, Lana J Williams, Felice N Jacka, Adrienne O’Neil, Julie A Pasco, Steven Moylan, Nicholas B Allen, Amanda L Stuart, Amie C Hayley, Michelle L Byrne, Michael Maes

**Affiliations:** 1 IMPACT Strategic Research Centre, School of Medicine, Deakin University, Geelong, VIC, Australia; 2 Department of Psychiatry, University of Melbourne, Parkville, VIC, Australia; 3 Florey Institute of Neuroscience and Mental Health, Parkville, VIC, Australia; 4 Orygen Youth Health Research Centre, Parkville, VIC, Australia; 5 School of Public health and Preventive Medicine, Monash University, Melbourne, VIC, Australia; 6 NorthWest Academic Centre, Department of Medicine, The University of Melbourne, St Albans, VIC, Australia; 7 Melbourne School of Psychological Sciences, University of Melbourne, Parkville, VIC, Australia; 8 Department of Psychiatry, Chulalongkorn University, Rama Road, Bangkok, Thailand

**Keywords:** Depression, Inflammation, Cytokines, Diet, Obesity, Exercise, Smoking, Vitamin D, Dental cares, Sleep, Atopic, Gut, Oxidative stress

## Abstract

**Background:**

We now know that depression is associated with a chronic, low-grade inflammatory response and activation of cell-mediated immunity, as well as activation of the compensatory anti-inflammatory reflex system. It is similarly accompanied by increased oxidative and nitrosative stress (O&NS), which contribute to neuroprogression in the disorder. The obvious question this poses is ‘what is the source of this chronic low-grade inflammation?’

**Discussion:**

This review explores the role of inflammation and oxidative and nitrosative stress as possible mediators of known environmental risk factors in depression, and discusses potential implications of these findings. A range of factors appear to increase the risk for the development of depression, and seem to be associated with systemic inflammation; these include psychosocial stressors, poor diet, physical inactivity, obesity, smoking, altered gut permeability, atopy, dental cares, sleep and vitamin D deficiency.

**Summary:**

The identification of known sources of inflammation provides support for inflammation as a mediating pathway to both risk and neuroprogression in depression. Critically, most of these factors are plastic, and potentially amenable to therapeutic and preventative interventions. Most, but not all, of the above mentioned sources of inflammation may play a role in other psychiatric disorders, such as bipolar disorder, schizophrenia, autism and post-traumatic stress disorder.

## Background

There is now an extensive body of data showing that depression is associated with both a chronic low-grade inflammatory response, activation of cell-mediated immunity and activation of the compensatory anti-inflammatory reflex system (CIRS), characterized by negative immunoregulatory processes [1,2]. New evidence shows that clinical depression is accompanied by increased oxidative and nitrosative stress (O&NS) and autoimmune responses directed against O&NS modified neoepitopes [3,4].

Not only is depression present in acute illness [4,5], but higher levels of inflammation appear to increase the risk for the development of *de novo* depression [6]. Indeed, cytokines induce depressive-like behaviors; in studies where healthy participants are given endotoxin infusions to trigger cytokines release, classical depressive symptoms emerge [7]. Exogenous cytokine infusions also cause the classical phenotypic behavioral and cognitive features of depression. As an exemplar, a quarter of the people given interferon for the treatment of hepatitis C develop emergent major depression [8,9]. Intriguingly, antidepressants, particularly selective serotonin reuptake inhibitors (SSRIs), *in vitro* or *ex vivo* exert significant negative immunoregulatory effects, decreasing the production of pro-inflammatory cytokines, for example, tumor necrosis factor (TNF)α and interleukin (IL)-1, T cell cytokines, for example, interferon (IFN)γ, and increasing that of anti-inflammatory cytokines, for example, IL-10 [10,11]. They additionally alter leucocyte mRNA gene expression of some immune markers. Galecki first documented altered expression of mRNA coding for cyclooxygenase-2, myeloperoxidase, inducible nitric oxide synthase and secretory phospholipase A2 type IIA in people with recurrent depressive disorder [12]. Additionally, inflammatory gene expression secondary to antidepressant therapy has been examined, with lowered levels of IL-1β and macrophage inhibiting factors seen after treatment, changes which were not associated with treatment response. However, lowering of IL-6 levels was associated with antidepressant response [13].

However, clinical depression is accompanied by a “resistance” to these *ex vivo* or *in vitro* effects of antidepressants attenuating inflammation and T cell activation [14]. Moreover, remission of clinical depression is accompanied by a normalization of inflammatory markers [15], while lack of response is associated with persistently elevated levels of inflammatory markers [16]. This resistance to the immunosuppressive effects of antidepressants in depressed patients may be explained by chronic inflammatory processes, chronic damage by O&NS and the onset of autoimmune responses [14].

These data beg the question: what are the sources of this chronic low-grade inflammatory and O&NS process and the source of the resistance to the well documented immunosuppressive effects of antidepressants? Any processes that activate chronic inflammatory and cell-mediated processes without a concomitant activation of the CIRS may further aggravate the detrimental effects of activated immuno-inflammatory pathways. It is well-known that many inflammatory disorders (chronic obstructive pulmonary disease, cardiovascular disease (CVD) and autoimmune disorders) and neuroinflammatory disorders (multiple sclerosis and Parkinson’s disorder) and inflammatory conditions (hemodialysis and the postpartum period) may trigger clinical depression [17]. However, these factors are only present in a small percentage of the larger population of depressed individuals. In contrast, there are a variety of widely prevalent environmental factors that are associated with increased risk for the development of depression. The aim of this review was, therefore, to collate extant data on the role of inflammation and O&NS as possible mediators of known environmental risk factors in depression, and to discuss potential implications of these findings, acknowledging the exploratory nature of these relationships. This paper will discuss those salient environmental variables that are risk factors for depression and examine immune dysregulation as a potential mediator of the interaction. This relationship has the potential to suggest both novel therapeutic and preventative approaches.

### Stress and trauma

Of all the factors in this review, stressors and trauma have attracted the greatest extant literature. Psychosocial stressors, including acute psychological trauma or more sub-chronic stressors, and early exposure to childhood trauma robustly increase the risk of developing clinical depression and mood symptoms, while impacting neuro-immune circuits. There is now evidence that in experimental animals, different types of psychosocial stressors increase systemic and CNS levels of pro-inflammatory cytokines, including IL-1 and IL-6. For example, immobilization stress, mild inescapable foot shock, chronic mild stress, tail restraint stress, and social isolation in rodent models cause significant increases in IL-1 (mRNA) levels in the plasma and brain [18-23]. Moreover, the onset of depressive-like behaviors following external stressors (for example, learned helplessness and chronic mild stress) is associated with activated transcriptional factors (for example, nuclear factor κB), activation of other inflammatory pathways (for example, cyclooxygenase 2 and prostaglandin production), and increased apoptosis (for example, lowered levels of Bcl-2 and Bcl-2-associated athanogene 1) [24].

In humans, there is evidence that different types of psychosocial stressors may stimulate the pro-inflammatory cytokine network, including increases in IL-6 and TNFα [25-28]. Maes *et al*. [28,29] were the first to report that stress-induced increases in IFNγ and stress-induced Th1 dominance were significantly correlated with stress-induced anxiety and distress. Thus, subjects with psychological stress-induced distress and anxiety showed significantly greater increases in IFNγ and lower IL-10 than those without distress and anxiety. Psychosocial stress is also accompanied by lowered levels of endogenous, anti-inflammatory compounds, for example, CC16 (uteroglobuline), which decreases the production of IFNγ [30]. Individuals showing stress-induced decreases in CC16 in the serum display higher stress-induced anxiety and distress, and an increased production of IFNγ during the stress condition [29,30]. Thus, stress-induced increases in pro-inflammatory and Th1-like cytokines may be mediated by lowered levels of endogenous anti-inflammatory compounds, such as CC16. Stress-induced production of pro-inflammatory cytokines, for example, TNFα and IL-6, and Th1-like cytokines, for example, IFNγ, are related to an increased number of leukocytes and neutrophils, and expression of immune cell activation markers, including CD2^+^CD26^+^ and CD2^+^HLADR, and different signs of an acute phase response [29]. This indicates that psychosocial stress-induced elevations in pro-inflammatory cytokines orchestrate stress-induced changes in peripheral blood immune cells, inflammatory reactions and neurobehavioral changes.

The findings that psychosocial stressors modulate the production of pro-inflammatory versus anti-inflammatory or negative immunoregulatory cytokines has important implications for stress-related disorders, including depression and post-traumatic stress disorder (PTSD). Thus, psychosocial stressors, such as negative life events, and chronic psychosocial stress often precede the onset of clinical depression. Translational models show that pro-inflammatory cytokines, such as IL-1β, IL-6 and TNFα, are depressogenic and anxiogenic. These mechanisms may explain why psychosocial stressors and acute psychotrauma may trigger mood disorders in vulnerable subjects, for example, those with immune gene polymorphisms, lowered levels of pepdidases, including dipeptidylpeptidase and prolylendopeptidase, and those with increased inflammatory burden [31].

Evidence from animal models has long suggested that early exposure to trauma in childhood may increase the subsequent risk of poor functioning of the immune, endocrine and nervous systems. More recently, studies conducted with humans have corroborated these findings. Data from the Dunedin Multidisciplinary Health and Development Study in New Zealand, a longitudinal study following 1,000 participants from birth to 32 years, have demonstrated that individuals experiencing stress in childhood resulting from maltreatment, abuse, social isolation and economic hardship are twice as likely to suffer chronic inflammation [32]. The detrimental impact of adversity on health in adulthood has also been demonstrated in US populations. Kiecolt-Glaser [33] found that childhood adversity can shorten the lifespan by 7 to 15 years, arguing that stress associated with abuse, death of a parent or parental relationship problems can lead to inflammation and premature cell aging, when compared with individuals who have not experienced such adversity. Miller *et al*. [34], in a further study focusing on depression outcomes, compared C-Reactive Protein (CRP) and IL-6 levels of women with and without history of childhood adversity; the former group was shown to have a greater likelihood of depression, recording higher levels of inflammation using these biomarkers. Studies exploring the influence of stress on other inflammatory diseases, such as CVD [35] and metabolic syndrome [36], have consistently shown similar trends. Such findings highlight the fundamental idea that stress occurring early in life can exert persistent effects over long periods of time, not only increasing susceptibility to somatic and psychiatric illness, but potentially interfering with treatment response.

However, the association between childhood adversity and vulnerability to inflammatory disease cannot fully be explained by a prolonged period of stress initiated by such an event. Rather, it is possible that learned, maladaptive responses to stress occurring in early childhood are also employed later in adult life in response to stressors. Thus, stress in adulthood has become of increasing interest as an instrumental risk factor for disease onset. For example, there is evidence that personality and the way in which an individual responds to psychosocial stressors, such as examination stress or job strain, may contribute to inflammatory processes [37]. Slavich *et al*. [38] found that responses to social stress via neural activity lead to marked increases in inflammatory activity. Similarly, Emeny [39] found job strain to have a direct effect on inflammation, and to influence other risk factors for inflammation. Job strain is known as a risk factor for other inflammatory diseases, such as CVD, and more recently has been shown to be strongly associated with depression risk [40]. Indeed, it is clear that understanding modifiable risk factors related to stress (and lifestyle) may be an important step in the prevention of inflammatory diseases like depression.

### Diet

There have been substantial changes to dietary habits globally over recent decades, wherein dietary patterns high in fiber, nutrient-dense foods and omega-3 poly-unsaturated fatty acids have been replaced by diets higher in saturated fats and refined sugars [41]. Whether diet quality contributes to psychopathology, particularly the common mental disorders (CMDs), depression and anxiety, has been a focus of much recent research. Since 2009, there have been numerous studies reporting inverse associations between diet quality and CMDs, both cross-sectionally [42-45] and prospectively [46-48]. These associations have also been shown in children [49] and adolescents [50-52] and are notably concordant across cultures. Individual nutrients are also related to depression. As an example, lowered availability of selenium in groundwater and lycopene contents in food are both associated with clinical depression [53-55].

One of the primary mechanisms of action proposed to explain these consistent relationships is that of inflammation, where diet quality can impact upon immune functioning and levels of systemic inflammation, which subsequently predisposes to depression. Data from population-based studies indicate an association between habitual diet quality and systemic inflammation. For example, in the Nurses’ Health Study, a healthy (‘prudent’) dietary pattern, characterized by higher intakes of vegetables and fruit, whole grains, fish and legumes, was associated with reduced plasma concentrations of inflammatory markers, including CRP and IL-6; conversely, an unhealthy (‘Western’) pattern, high in red and processed meats, refined carbohydrate and other processed foods, was associated with increased inflammatory markers [56]. Similarly, Fung *et al*. [57] found that a Western dietary pattern was associated with higher levels of CRP in men participating in the Health Professionals Follow-up Study, while in the ATTICA study, a Mediterranean diet pattern was associated with lower inflammatory markers [58].

Various components of diet may also influence inflammation. For example, the fiber contained in whole grain foods appears to have immune modulating functions; wholegrain foods are rich in beta-glucans and these are known to promote immune functioning [59]. Fiber influences gut microbiota [60], and this has a knock-on effect on immune functioning [61]. In support of this, the consumption of whole grains is shown to be inversely associated with death from non-cardiovascular, non-cancer inflammatory diseases [62]. Whole grain foods are also high in phytochemicals, which protect against the oxidative stress that is a consequence of inflammation and a feature of depressive illness [63]. High glycemic load (GL) diets are a common feature of Western culture, being heavy in refined carbohydrates and added sugars. In middle-aged, otherwise healthy, women, a high GL diet was shown to be associated with higher levels of CRP [64], while another larger study reported that a high glycemic index diet was associated with a small but significant increase in CRP in more than 18,000 middle- to older-aged women [65]. Omega-3 fatty acids, which are important components of many healthy foods, such as seafood, nuts, legumes and leafy green vegetables, act to reduce inflammation [66], while a diet disproportionately high in omega-6 fatty acids, which are commonly used in the production of processed foods, increases the production of pro-inflammatory cytokines [67]. In the Whitehall II cohort study, polyunsaturated fatty acid levels were inversely associated with CRP, while higher saturated fatty acid levels in serum phospholipids were associated with higher CRP and fibrinogen [68]. Trans-fatty acids similarly induce inflammation [69]. Finally, magnesium intake, which is highly correlated with diet quality [43], was shown to be inversely associated with CRP levels in the large National Health and Nutrition Survey (NHANES) in the US [70].

Intervention studies in humans support these observational data. Men randomized to a diet high in fruits and vegetables (eight servings per day) for eight weeks demonstrated a significant decrease in CRP compared with those consuming only two servings per day [71]. Similarly, Jenkins *et al*. [72] reported that a dietary intervention using a whole-diet approach and emphasizing the intake of soy, nuts and plant foods, resulted in pronounced reductions in CRP levels in hyperlipidemic patients over one month, independently of changes in body weight. Esposito *et al*. [73] also reported reductions in multiple inflammatory markers in patients with the metabolic syndrome randomized to a Mediterranean-style diet, long recognized as a healthful dietary pattern, independent of observed decreases in weight. Conversely, in an intervention study of overweight adults, a sucrose-rich diet for 10 weeks resulted in significant increases in the inflammatory markers haptoglobin and transferrin, and small increases in CRP [74].

Finally, studies in animal models explicate specific mechanisms of action. Recent studies show that rodents maintained on diets high in saturated fatty acids have elevated markers of brain inflammation [75]. This effect appears to be trans-generational; rats born to dams fed high saturated fat or high trans-fat diets were shown to have increased levels of neuroinflammation in adulthood, even when fed a standard diet post-weaning [76]. Saturated and trans-fat intake may influence inflammation, at least in part, via the health of the gut. High fat intake increases elements from gut microbiota, such as the endotoxin lipopolysaccharide (LPS), in the circulatory system, and LPS are potent promoters of immune system activation [77]. However, some of these deleterious effects on immune functioning may be addressed through the consumption of certain types of resistant starches and prebiotics [78]. In particular, short-chain fatty acids (SCFAs), which are produced by fermentation of dietary fiber by intestinal microbiota, appear to have a positive impact on immune functioning, suggesting that increasing intake of fermentable dietary fiber may be important in reducing inflammation [79]. There is an increasing focus on the importance of gut microbiota in depression and this is addressed in further detail below.

### Exercise

There is a substantive evidence base on the role of exercise as an effective treatment strategy for depression [80,81]. It is also evident that habitual or regular exercise protects against the development of new depressive illnesses [82-84], and that physical inactivity during childhood is associated with an increased risk of depression in adulthood [85]. In a nested case-control study of older individuals, habitual physical activity reduced the likelihood of new depressive and anxiety disorders; for each standard deviation increase in physical activity score, there was a halving in the likelihood of developing depressive or anxiety disorders [82]. The relationship in this, and other studies [86-88], was found to be driven by leisure-time physical activity. Resistance training is a recognized treatment strategy for slowing loss of skeletal muscle mass and function [89]. A prospective cohort study in Tasmania reported that leisure-time physical activity is positively associated with leg strength and muscle quality in older women [90]. Sarcopenia is linked to elevated high sensitivity (hs) CRP [91], especially in the presence of obesity. Sarcopenia is further linked to cognitive decline in the elderly, which appears to be mediated by inflammation [92].

Acute exercise generates *reactive oxygen species* (ROS) [93] and inflammatory cytokines [94] that can transiently damage muscle cells, causing muscle fatigue, pain and inflammation. Contracting skeletal muscle produces a number of ‘myokines’, such as IL-6 [95], which impact systemically on lipid and glucose metabolism [96]. The pattern of inflammatory markers produced during acute exercise, characterized by a rapid elevation in levels of IL-6 that is quickly followed by induction of anti-inflammatory substances, including IL-1ra, IL-10 and soluble tumor necrosis factor receptor (sTNF-R) [97], differs markedly from that in other inflammatory conditions, such as sepsis. Recovery after the exercise-induced IL-6 spike dampens the inflammatory response and oxidative burst activity [98]. Chronic or regular exercise, therefore, down-regulates systemic inflammation via homeostatic adaptation [99]. Similarly, fitness and exercise reduces leptin [100], elevated levels of which are also implicated in the development of depression [101] and is the most evidence-based management strategy for insulin resistance [102]. These data converge to provide evidence supporting a role for inflammation in exercise-induced mood improvements.

More recently and conversely to the association between inflammation and exercise, the relationship between sedentary behavior and inflammation has become of increasing interest. Sedentary behavior is now considered an important and novel risk factor for a number of physical health conditions, independent of moderate to vigorous physical activity levels. Specifically, sedentary behavior has been shown to be associated with elevated adiposity and cardiovascular risk. For example, in a multi-ethnic study of atherosclerosis Allison *et al*. (2012) found sedentary behavior to be linked with “unfavorable” levels of adiposity-associated inflammation [103]. Further, in a national survey conducted in the US, Koster *et al*. [104] found sedentary behavior to be a predictor of mortality, after adjustment for relevant covariates. Complicating interpretation is that factors that are predictive of lower physical activity, such as lower self-efficacy, medical co-morbidity, lower educational status and social isolation, may be mediators or moderators of the association [105]. While the underlying physiology associated with inactivity is also not fully understood, there is evidence from animal studies that a sedentary lifestyle may suppress skeletal muscle lipoprotein lipase [106]; responsible for controlling the process associated with metabolic risk factors. Further research is required in order to fully understand the links between inflammation and the underlying physiology of sedentary behavior.

### Obesity

Closely linked to diet are its consequences, including obesity, which is a growing public health concern linked to a host of chronic physical health conditions [107]. With the prevalence of obesity increasing to epidemic proportions, efforts in understanding associated risk factors and outcomes are continuing. The most recently collected data have shown that in excess of 60% of the Australian population exceed the recommended threshold for healthy body habitus [108]; concordant with estimates from other countries [109]. With few exceptions, both clinical- and community-based cross-sectional studies have consistently shown a relationship between obesity and depression regardless of methodological variability [110,111]. Prospective studies have suggested that obesity may be a clinical condition that predisposes to the development of depressive symptomatology as well as clinical depression [112]. Depression has also been shown to predispose to obesity in a bidirectional manner [112]. A recent meta-analysis of prospective cohort studies found obesity to increase the risk of later depression by 55%, while depression increased the risk of developing obesity by 58% [113]. Further investigations into mechanistic pathways are much needed.

Obesity is an inflammatory state. Inflammatory cytokines have been found in abundance in fat cells, are involved in fat metabolism and have been observed to be positively associated with all indices of obesity, in particular abdominal obesity [114]. Altered adipocyte function, fatty acid levels, leptin and hypothalamic pituitary adrenal (HPA) axis dysfunction and oxidative stress are hypothesized to play a crucial but synergistic role in obesity-associated inflammation [114]. A reduction in adipose tissue mass, through calorie restriction in a group of obese women, was shown to reduce the ability of adipose tissue to produce TNFa, IL-6, IL-8 and leptin [115]. Cross-sectional and prospective studies indicating obesity, independent of age and other potential confounders, leads to altered levels of inflammatory cytokines (or vice visa) provides a likely explanation into the observed increases in concomitant disease, including depression [116,117]. Moreover, we and others have previously shown inflammation, in particular, serum hsCRP to predict *de novo* major depressive disorder (MDD) [6].

### Smoking

Rates of cigarette smoking are significantly higher in patients experiencing depression when compared with non-depressed controls. This finding has been replicated in numerous population-based epidemiological studies [118,119]. The causal relationship between smoking and depression is, however, a complex one. The three potential causal connections underpinning the cross-sectional relationship, that smoking leads to depression [120,121], that depression increases smoking behaviors [122], and that shared-vulnerability factors [123] increase the risk of both, are all supported by empirical evidence. Although it is probable that cigarette smoking exerts diverse psychological and neurobiological effects, which may increase one’s predisposition to developing depression, one major pathway could be through enhancing systemic inflammatory and cell-mediated immune responses, and enhancing exposure to O&NS.

Cigarette smoke contains many thousands of chemicals [124], including free radicals, metals, tars and other substances that induce inflammatory responses in bodily tissues and increase levels of O&NS. The noxious effects of cigarette smoking in inducing altered inflammatory responses contribute to a number of chronic physical illnesses, including asthma, chronic obstructive pulmonary disease and atherosclerosis [125-127]. Smoking has been associated with increased levels of acute phase proteins, including CRP, and pro-inflammatory cytokines, including IL-1β, IL-6 and TNF–α, which occur secondary to direct effects in activation of microglia and astrocytes [128]. These findings of increased pro-inflammatory cytokines are similar to those found in depressed patients [3]. Recent evidence also suggests that enhanced inflammatory responses are additive between cigarette smoking and depression, such that depressed smokers exhibit higher levels of hsCRP, IL-6 and TNF –α than non-depressed smokers [129].

The exogenous free radicals contained in cigarette smoke lead to direct oxidative damage to cellular tissues, including those in the CNS. Numerous studies have demonstrated that animals exposed to cigarette smoke exhibit increased markers of oxidative stress and decreased levels of antioxidants. Observed effects include increased levels of thiobarbituric acid reactive substances (TBARS), superoxide, carbonylated proteins [130] and measures of lipid peroxidation [131-133], and reductions in levels of antioxidant enzymes, such as catalase [134], glutathione, superoxide dismutase [134], glutathione reductase, glutathione peroxidase and Vitamins A, C and E [135]. These findings appear most evident in models of chronic cigarette exposure, suggesting the possibility that early adaptive responses [136], which may increase antioxidant levels in the short term [137], are overwhelmed by chronic use. Once again, these findings are similar to those found in patients in major depression, where there appears to be a disturbance in the oxidant/antioxidant balance [3].

Significant interaction occurs between markers of inflammation and O&NS, which further interact with numerous other key elements of central nervous system functioning, including neurotransmitter systems, neuroplastic neurotrophins, mitochondrial energy production and epigenetic controls. Through these diverse effects, in conjunction with its known ability to increase inflammatory and oxidative stress responses, cigarette smoking may increase susceptibility for the development of depression. The extent to which the susceptibility is increased will likely differ between individuals based on underlying depression risk, differing levels and timing of exposure to cigarette smoke (for example, childhood versus adulthood) and presence and severity of cigarette-related health and social consequences.

### Gut permeability, the microbiome and the toll-like receptor (TLR)-IV pathway

A new potential pathway that may mediate depression pathogenesis is increased immune responses against LPS of different commensal, gram negative bacteria. Clinical depression has recently been shown to be accompanied by increased plasma levels of immunoglobulin (Ig) A and/or IgM directed against a number of gram negative bacteria, including *Hafnia alvei*, *Pseudomonas aeruginosa*, *Morganella morganii*, *Proteus mirabilis*, *Pseudomonas putida*, *Citrobacter koseri* and *Klebsielle pneumoniae*[138-140]. All these gram negative bacteria belong to the normal gut flora [141,142]. These results suggest that there is an IgA- and IgM-mediated immune response directed against LPS, which is part of the bacterial wall of gram negative bacteria. LPS are toxic substances, which may activate immune cells by binding to the CD14-Toll-like receptor-4 (TLR4) complex. This in turn may activate intracellular signaling molecules, such as nuclear factor (NF)-κβ, which in turn activates the production of pro-inflammatory cytokines, including TNFα and IL-1 and cyclo-oxygenase-2 (COX-2) [143,144]. The same processes also induce O&NS pathways, for example, increased expression of inducible nitric oxide (iNOS) and thus NO [143]. LPS further activates nicotinamide adenine dinucleotide phosphate (NADPH) oxidase leading to an increased production of ROS, for example, peroxides, and superoxide [145,146]. Moreover, LPS increases the production of lysozyme (muramidase), which is produced by neutrophils, monocytes and glandular cells and which may bind LPS and therefore may decrease the activities of LPS [147].

The systemic IgM-mediated immune response in depression directed against LPS suggests that bacterial translocation may play a role in the inflammatory and O&NS pathophysiology of clinical depression. Bacterial translocation indicates the presence of “leaky gut” or an increased permeability of the gut wall or loosening of the tight junction barrier. Under normal conditions, immune cells are geographically separated from gram negative bacteria in the gut. An increased permeability of the gut wall may allow poorly invasive gram negative bacteria to translocate into the mesenteric lymph nodes (MLNs) and sometimes into the systemic circulation [148,149]. Consequently, in the systemic circulation, IgM and IgA responses are mounted against the LPS of the bacterial wall, while IgA responses may be mounted even when the bacteria do not reach the blood stream, but only translocate into the MLNs. Thus, the assay of the IgA responses directed against LPS measures bacterial translocation into the blood stream and the MNLs. Once primed, immune cells may produce pro-inflammatory cytokines and stimulate O&NS pathways [140]. Elevated plasma levels of IgA and IgM levels directed against the LPS of gram negative commensals indirectly indicate increased bacterial translocation and thus increased gut permeability. Therefore, bacterial translocation may drive inflammatory and O&NS processes in depression, even in the absence of a specific inflammatory lesion [138]. On the other hand, inflammatory and O&NS pathways may cause loosening of the tight junction barrier through NF-κB and pro-inflammatory cytokine-related mechanisms [150-154].

In a recent study, the IgM and/or IgA responses directed against LPS were found to be associated with signs of inflammation, O&NS processes and even autoimmune responses [140]. More specifically, increased IgM and IgA responses to LPS in depression are significantly and positively correlated to plasma lysozyme, serum oxidized LDL antibodies and the IgM responses directed against azelaic acid and malondialdehyde and phosphatidylinsositol, and NO-adducts, such as NO-tryptophan and NO-tyrosine [140]. These findings not only highlight O&NS processes, but also oxidative damage to lipids and nitrosative damage to proteins, and autoimmune responses mounted against neoepitopes formed by O&NS damage to lipids and proteins [140].

Thus, increased bacterial translocation may be a primary factor in the onset of clinical depression and may be a secondary factor further aggravating inflammatory and O&NS pathways, leading to a vicious cycle between loosening of the tight junction barrier and activation of inflammatory and O&NS pathways [138]. In addition, the IgM responses directed against LPS were significantly higher in patients with chronic depression than in those without chronic depression [155]. This may suggest that the inflammatory, O&NS and autoimmune processes that are induced by bacterial translocation could be involved in the development of chronic depression and the neuroprogression that is observed in this condition [3,4,139]. Recently, translational data further underscored the importance of increased gut permeability in mediating stress-related behavioral responses, including depression [156]. Thus, stress activates the TLR-IV pathway and associated inflammatory and O&NS pathways, including central neuroinflammation. These effects are at least in part mediated by stress-induced intestinal permeability and bacterial translocation [156].

### Atopic disorders

An elevated IgE response to common allergen exposure, leading to the development of allergic symptoms, such as asthma, eczema or allergic rhinitis/hay fever is defined as atopy [157]. The prevalence of atopic disorders has been steadily increasing over the past few decades [158,159]. Interestingly, atopy and depression have recently been linked. Although methodologies differ among studies, it has been consistently reported that atopic disorders are associated with an increased risk of both clinical depression and depressive symptomatology in clinical settings [160-163]. Population-based studies provide further support, showing a positive association between depression and atopic disorders [164-168]. As with all of the associations explored in this paper, the causal pathways and their mediators merit exploration.

Atopic disorders are the product of an inflammatory response. The interaction of an antigen, with antigen-specific IgE antibodies fixed on the mast cell surface, activates the mast cell to produce the release of inflammatory mediators [169]. There are three categories of mediators released; secretory granule-associated mediators (for example, histamine, proteoglycans, neutral proteases), lipid-derived mediators (for example, cycloxygenase and lipoxygenase metabolites of arachidonic acid) and cytokines (for example, Th2 response IL4, IL5 and IL13 and TNFa) [170]. This response results in an immediate hypersensitivity reaction, such as edema or itch of the skin, cough or bronchospasm, sneezing or increased mucous secretion. Many hypersensitivity reactions result in a second reaction, termed the late phase reaction (for example, persistent asthma) [169,170].

### Dental cares and periodontal diseases

Dental cares and periodontal diseases, including gingivitis and periodontitis, are diseases of the oral cavity where connective gum tissue gradually becomes detached from the alveolar bone and often leads to tooth loss [171]. Periodontal disease is a considerable public health concern; a recent prevalence estimate in US adults was 47% [172]. Correlates of periodontal disease include psychological factors, such as low self-esteem [173], loneliness [174] and high levels of stress [175]. It has been reported that psychiatric patients have poorer oral health status [176]. Recent research suggests that depression in particular may be associated with periodontal disease. For example, a large, epidemiological study of over 80,000 adults found that adults with depression were less likely to use oral health services, and adults with anxiety or depression were more likely to have tooth loss, even after controlling for various demographic and health factors, including use of oral health services [177]. However, another study comprising an older population found no association between depression and any measure of oral health, including periodontal disease [178]. Much of the limited research on psychological factors and periodontal disease examines samples from specialist or patient populations. Therefore, research which focuses on correlates of oral health and depression from community samples that are more representative of the general population, and that examines pathways and mediators of this association, are required.

Periodontal disease is an inflammatory disease. The accumulation of bacterial plaque on the teeth causes lesions in the periodontal tissue, leading to an acute, local inflammatory response [179]. Local inflammation in gingivitis is concentrated in soft oral tissues, such as the gum and connective tissue, while inflammation in supporting structures, including the alveolar bone, is also present in periodontitis [180]. Critically, periodontal disease is also associated with high levels of systemic inflammation, such as elevated serum levels of CRP [181]. Furthermore, it is a significant predictor of other inflammatory illnesses, such as CVD [182], and health outcomes, such as mortality in diabetes [183] and coronary artery disease [184]. The inflammatory response resulting from periodontal disease appears to be mediated by macrophages, which produce various cytokines [185], although periodontal tissues may also directly produce cytokines, such as IL-6 and IL-8 [186]. As such, periodontal disease may be a marker of a failure of the immune system to resolve inflammation [187,188], a state that may also result in vulnerability to depression [189]. Furthermore, there may also be direct causal links between depression and periodontal disease, such as when periodontal disease increases risk for depression through the psychosocial effects of poor oral hygiene (for example, shame, isolation, loneliness) or more directly through the systemic inflammatory effects of periodontal disease that may potentiate inflammatory and O&NS processes and thus depressive symptoms. Currently, there remains a dearth of evidence that examines whether translocation of periodontal bacteria plays a role in some patients with clinical depression, despite some evidence that periodontal infections may play a role in neurodegenerative disorders [190].

### Sleep

Sleep is one of the most widely observed phenomena in multi-cellular organisms [191] and is recognized to play a vital regulatory role in a number of physiological and psychological systems. Abnormal sleep patterns are associated with a number of adverse health outcomes, such as an increased risk for mortality [192], morbidity and poorer quality of life [193]. Sleep disturbance is a common element in psychiatric disorders, and a complimentary marker of psychopathology in mood disorders [194]. It is estimated that up to 80 to 90% of individuals who suffer from a MDD also experience sleep disturbances [194-196]. Typically, depressive patients exhibit higher rates of sleep disturbances than those in the general population [197] and, conversely, those who report abnormal sleep patterns report higher levels of depression than normal sleepers [198]. Several prospective and epidemiological studies have suggested that sleep disturbances may also predispose individuals to subsequent development of mood disturbances. Indeed, a meta-analysis comprising relevant longitudinal epidemiological studies conducted by Riemann and Volderholzer [199] concluded that insomnia symptoms unambiguously represented a risk factor for the later development of depression. Similar research has suggested that insomnia symptoms often increase the risk of relapse in individuals previously diagnosed with MDD [200], and that periods of sleeplessness often precede manic episodes in bipolar patients [201].

Both chronic and acute sleep deprivation are associated with alteration in cellular and natural immune functioning [202]; however, the direct mechanism by which sleep affects inflammation is unclear. It is thought that alterations in sleep as a result of lifestyle or medical factors act as a moderator for inflammatory biomarkers [203] via a bidirectional relationship that exists to modulate host-defense and sleep mechanisms [192]. Experimental research has demonstrated that acute sleep deprivation results in impairments in immune functioning [202], characterized by increased levels of the pro-inflammatory cytokines, CRP, TFN-α [204] and IL-6 [205]. These alterations contribute to stroke and heart attack due to long-term impaired vascular endothelial function [206] and possible renal impairment [207]. Even modest sleep restriction (from eight to six hours per night) has been shown to result in elevation in levels of IL-6 and TFN-α [208]; however, this has not been replicated in epidemiological studies [209]. Increases in these biomarkers have also been observed naturally in individuals suffering primary insomnia [208,210]. Activation of these pro-inflammatory pathways may result from increased nocturnal sympathetic arousal [193] and an associated decline in natural immune functioning [202], therefore, facilitating potentially poorer cardiovascular outcomes and higher mortality risks previously seen in these individuals [192,211].

Growing research has suggested that curtailment of sleep is associated with similar neuroendocrine and neurobiological abnormalities observed in mood disturbances [212]. Increases in pro-inflammatory cytokines TFN-α and IL-6 following sleep deprivation are also thought to be related to a reduction in adult neurogenesis (AN), comparable to those disturbances found in depressive patients [213]. Cytokines are significant modulators of mood (Krishnan and Nestler, [214]). The release of low doses of IL-6 and TFN-α via administration of IL-1 in rats generates ‘sickness behavior’ (social withdrawal, decreased exploratory behavior) [2,215], while deletion of the gene encoding IL-6 or TFNα promotes antidepressant-like behavior phenotypes (resistance to helplessness, enhanced hedonic behavior) [216]. Increased activation of the immune system is often observed in depressed patients; and those suffering immune diseases often report higher rates of depression [215]. It has, therefore, been proposed that inhibition of neurogenesis through the process of chronic sleep disruption may also contribute to the etiology of depression [217]. As both improved nocturnal sleep and successful pharmacological treatment of depression are associated with decreased levels of IL-6 [208,218], and similar inflammatory mechanisms appear to contribute to the pathogenesis of depression and expression of illness in chronic sleep disordered patients, adaptive sleep habits may, therefore, act as a protective factor against cardiovascular risk and poorer mental health outcomes.

### Vitamin D

Low levels of Vitamin D, particularly 25-hydroxyvitamin D are widespread among Western populations [219], making it the most prevalent deficiency state. Low Vitamin D is linked to a diversity of adverse health outcomes, such as osteoporosis and cancer [220]. Notably, the physiology of vitamin D overlaps with the pathophysiology of depression. Vitamin D receptors are expressed in key brain areas; and vitamin D has a role in circadian rhythms and sleep, affects glucocorticoids and influences neuronal growth, cell proliferation in the developing brain and embryogenesis [221]. There is a growing epidemiological evidence-base linking depressive symptoms to low levels of serum 25-hydroxyvitamin D. These studies include both cross-sectional studies, as well as prospective data suggesting that low levels are associated with increased risk for the development of depression. There are positive trials of the potential antidepressant effects of vitamin D [222], although there are equally negative trials [223].

Vitamin D has well documented modulatory effects on immunity. It modulates immune responses to infections, such as tuberculosis [224]. In rats given a high fat diet, 1α, 25-dihydroxyvitamin D3 (calcitriol) treatment reduced concentrations of various inflammatory markers, including TNF-α, CRP and IL-6, and protected the liver from inflammatory damage [225]. In human studies, supplementation robustly reduces inflammatory markers in people with cystic fibrosis, including TNF-α and IL-6, but not other cytokines. Curiously, those two cytokines are the most robustly associated with depression in meta-analyses [226]. In multiple sclerosis, vitamin D reduces markers of inflammation and attenuates disease progression [227]. A one-year clinical trial of supplementation with Vitamin D in obese individuals reduced TNF-α levels, but increased hsCRP. The implications of these changes are unclear [225]. Inflammation and oxidative stress are tightly interlinked, and in human studies, vitamin D supplementation additionally reduced oxidative stress markers [228]. Vitamin D is a proxy of sunlight exposure, and it is useful to note that sunlight may suppress immunity via pathways other than via vitamin D. In fact, vitamin D derived from safe sunlight exposure may reduce systemic inflammation. There are additional skin photoreceptors that absorb ultraviolet light, and play a role in immunoregulation, that include DNA and lipids in skin cells and *trans*-urocanic acid found in the stratum corneum [229].

### Inflammation and immune activation across major psychiatric disorders

There is also evidence that many other major psychiatric disorders are accompanied by activation of inflammatory and cell-mediated immune pathways, for example, mania, schizophrenia, post-traumatic stress disorder (PTSD). The first papers showing inflammation (increased levels of pronflammtory cytokines, such as IL-6 and acute phase proteins; [230,231] and immune activation (increased levels of sIL-2Rs levels [230,232] in acute and euthymic manic patients were published in the 1990s. A recent meta-analysis confirmed that mania and bipolar disorder are accompanied by activation of inflammatory, cell-mediated and negative immunoregulatory cytokines [233]. Based on the first results obtained in schizophrenia, Smith and Maes in 1995 launched the monocyte-T lymphocyte theory of schizophrenia, which considered that activation of immuno-inflammatory processes may explain the neurodevelopmental pathology related to gestational infections. Results of recent meta-analyses showed that schizophrenia is accompanied by activation of inflammatory and cell mediated pathways [234]. PTSD patients also show higher levels of pro-inflammatory cytokines, including IL-1 [235], IL-6 [236,237] and TNFα [238].

It is evident that the sources of inflammation and immune activation, which play a role in depression, may contribute to the inflammatory burden in patients with mania. Schizophrenia is also associated with some but not all sources of inflammation and immune activation that play a role in depression. For example, a recent review showed that stress and trauma (first and second hits), nutritional factors and vitamin D may play a role in schizophrenia [239]. The strong associations among schizophrenia and smoking [240], obesity [241], some atopic disorders [242], sleep disorders [243] and poor periodontal and oral health [244,245] may further contribute to the inflammatory burden in schizophrenia patients. Other factors, however, may be more specific to mood disorders than to schizophrenia. For example, there is no significant association between schizophrenia and increased bacterial translocation [Maes *et al*., personal data]. There is strong comorbidity between depression and PTSD and patients with this comorbidity show increased inflammatory responses as compared with those with PTSD or depression alone [236,237]. The severity of stress and trauma [236], and the association between PTSD and smoking [246], obesity/metabolic syndrome [247], oral health status [248] and sleep disorders [249] may further aggravate the activation of immuno-inflammatory pathways in PTSD or comorbid PTSD and depression.

## Summary

In interpreting these data, a number of factors need to be borne in mind. First, depression is a very pleomorphic and heterogeneous phenotype, and there are likely to be substantial differences in results depending whether studies examine clinical or non-clinical samples, use cut scores on rating scales or formal structured interviews and so on. Similarly, many studies do not control for potential confounders, and most of the literature is cross-sectional. Last, the areas of interest diverge greatly in terms of the quantity and quality of the extant literature, with a clear picture emerging on some areas, such as trauma and stress, and others remaining areas for future investigation.

The identification of a number of potential factors that are known sources of inflammation, and their correlation to quality evidence linking those factors to increased risk of depression, provides mechanistic support for inflammation as one of the mediating pathways to both risk and neuroprogression in depression. The pivotal element is that most of these are plastic, and amenable to intervention, both therapeutic and preventative. While inflammation has suggested a number of very promising anti-inflammatory therapies, including statins, aspirin, pioglitazone and celecoxib, the latter preventative need is perhaps the more pressing [14,250,251]. Psychiatry largely lacks an integrated model for conceptualizing modifiable risk factors for depression. It has, therefore, lacked conceptually and pragmatically coherent primary prevention strategies, prioritizing the treatment of established disorders. Yet the rationale, targets and imperative to focus on prevention of depression at a population level is clear.

## Abbreviations

CIRS: Compensatory anti-inflammatory reflex system; CMDs: Common mental disorders; CNS: Central nervous system; COX-2: Cyclo-oxygenase-2; CRP: C-reactive protein; CVD: Cardiovascular disease; HPA axis: Hypothalamic pituitary adrenal axis; hs: High sensitivity; IFN: Interferon; Ig: Immunoglobulin; IL: Interleukin; iNOS: Inducible nitric oxide; LPS: Lipopolysaccharide; MDD: Major depressive disorder; MLNs: Mesenteric lymph nodes; NADPH: Nicotinamide adenine dinucleotide phosphate; NHANES: National Health and Nutrition Survey; NF: Nuclear factor; O&NS: Oxidative and nitrosative stress; PTSD: Post-traumatic stress disorder; ROS: Reactive oxygen species; SCFAs: Short-chain fatty acids; SSRIs: Selective serotonin reuptake inhibitors; sTNF-R: Soluble tumor necrosis factor receptor; TNF: Tumor necrosis factor; TBARS: Thiobarbituric acid reactive substances; TLR: Toll-like receptor.

## Competing interests

MB has received Grant/Research Support from the NIH, Cooperative Research Centre, Simons Autism Foundation, Cancer Council of Victoria, Stanley Medical Research Foundation, MBF, NHMRC, Beyond Blue, Rotary Health, Geelong Medical Research Foundation, Bristol Myers Squibb, Eli Lilly, Glaxo SmithKline, Organon, Novartis, Mayne Pharma and Servier; has been a speaker for Astra Zeneca, Bristol Myers Squibb, Eli Lilly, Glaxo SmithKline, Janssen Cilag, Lundbeck, Merck, Pfizer, Sanofi Synthelabo, Servier, Solvay and Wyeth; and served as a consultant to Astra Zeneca, Bristol Myers Squibb, Eli Lilly, Glaxo SmithKline, Janssen Cilag, Lundbeck Merck and Servier.

FJ has received Grant/Research support from the Brain and Behaviour Research Institute, the National Health and Medical Research Council (NHMRC), Australian Rotary Health, the Geelong Medical Research Foundation, the Ian Potter Foundation, Eli Lilly and The University of Melbourne, and has been a paid speaker for Sanofi-Synthelabo, Janssen Cilag, Servier, Pfizer, Health Ed, Network Nutrition and Eli Lilly. She is currently supported by an NHMRC Training Fellowship (#628912).

LW, JP, SM, AH and MM have no conflicts of interest, including specific financial interests and relationships and affiliations relevant to the subject matter or materials discussed in the manuscript.

## Authors’ contributions

MB took part in the conception and design of the study, critically revised the manuscript and took primary responsibility for writing the manuscript. LW, FJ, AO, JP, SM, NA, AS, AH, MLB and MM took part in writing the manuscript and critically revised the manuscript. All authors read and approved the final manuscript.

## Pre-publication history

The pre-publication history for this paper can be accessed here:

http://www.biomedcentral.com/1741-7015/11/200/prepub
